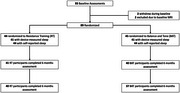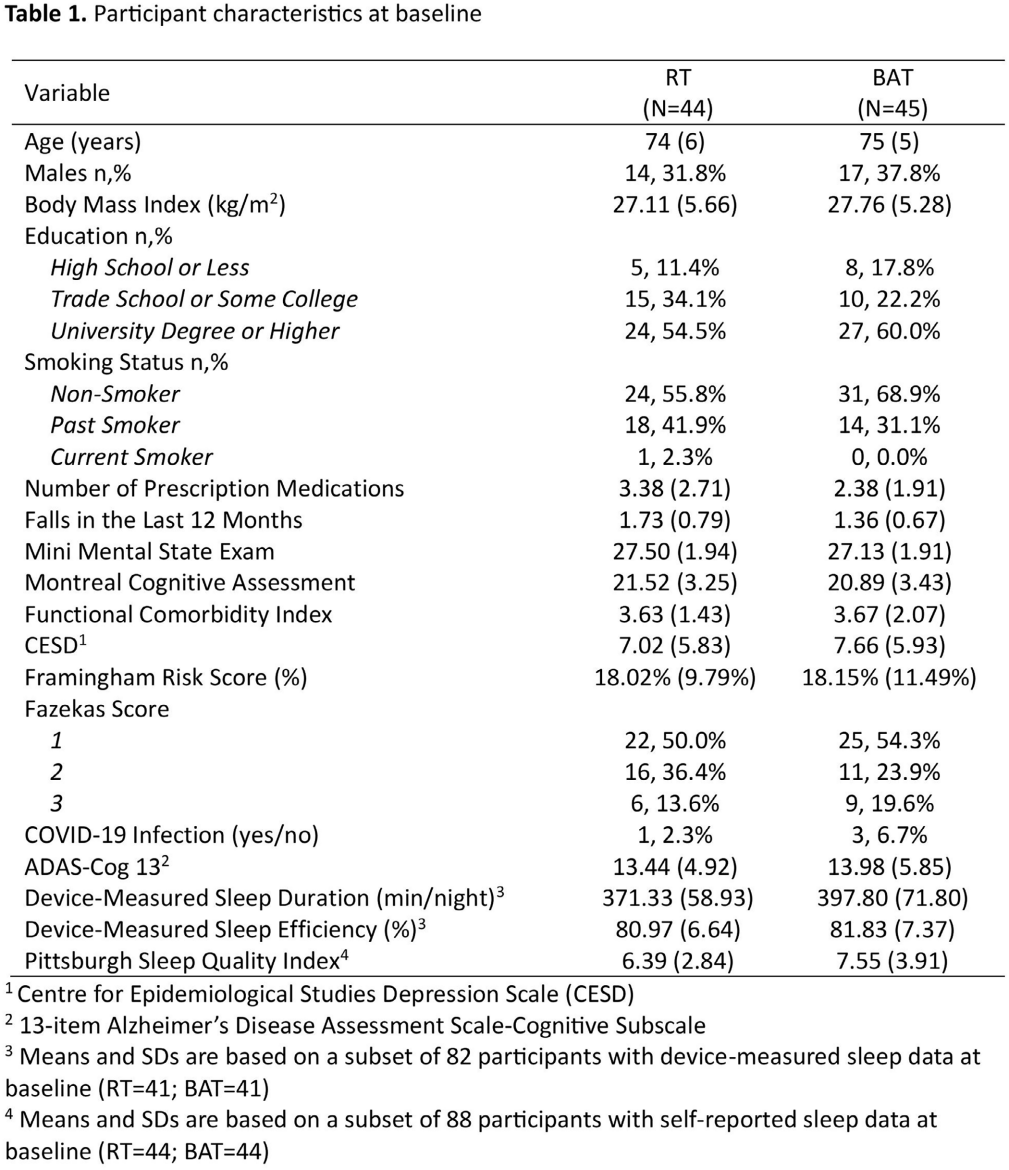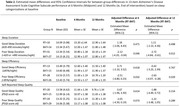# Sleep moderates the effects of resistance training on cognition among older adults with subischemic vascular cognitive impairment: Secondary results of a randomized clinical trial

**DOI:** 10.1002/alz70860_097971

**Published:** 2025-12-23

**Authors:** Ryan S Falck, Ryan G Stein, Rachel A Crockett, Roger C. Tam, Teresa Liu‐Ambrose

**Affiliations:** ^1^ Centre for Aging SMART, Vancouver Coastal Health Research Institute, Vancouver, BC, Canada; ^2^ Djavad Mowafaghian Centre for Brain Health, Vancouver, BC, Canada; ^3^ Vancouver Coastal Health Research Institute, Vancouver, BC, Canada; ^4^ University of British Columbia, Vancouver, BC, Canada; ^5^ University of Oxford, Oxford, United Kingdom

## Abstract

**Background:**

Subcortical ischaemic vascular cognitive impairment (SIVCI) is the most common cause of vascular cognitive impairment. Resistance training (RT) is a promising strategy to promote cognitive function among adults with SIVCI. Emerging evidence suggests sleep may be a key moderator of the effects of exercise on cognitive function. Thus, we examined whether sleep moderates the effects of RT on cognitive function in individuals with SIVCI.

**Method:**

A 12‐month, parallel group, secondary analysis of a randomized controlled trial (RCT) among community‐dwelling adults with SIVCI, aged 55+ years. Participants were randomly allocated to receive 12 months of either 1) twice‐weekly progressive RT or 2) twice‐weekly balance and tone (BAT). At baseline, device‐measured sleep duration and efficiency were indexed using wrist‐worn actigraphy; self‐reported sleep quality was measured by Pittsburgh Sleep Quality Index (PSQI). Participants were classified at baseline as having good or poor device‐measured duration, device‐measured efficiency, or self‐reported quality based on PSQI. Cognitive function was indexed using the 13‐item Alzheimer's Disease Assessment Cognitive Subscale (ADAS‐Cog 13) at baseline, 6 months (midpoint), and 12 months (end of intervention). We examined if baseline sleep categorizations (i.e., good/poor) moderated the effects of RT on ADAS‐Cog 13.

**Result:**

Eighty‐nine participants were randomized (Figure 1; RT=44; BAT=45). Participants are described in Table 1. Mean age was 75 years (SD=6 years) and 65.2% were female. At baseline, mean sleep duration was 385 minutes/night (SD=67 minutes/night) and mean efficiency was 81.40% (SD=6.98%); average ADAS‐Cog 13 score was 13.71 (SD=5.38). Table 2 describes our moderation analysis. Compared with BAT participants with poor sleep duration, RT participants with poor sleep duration had better ADAS‐Cog 13 performance following the intervention (estimated mean difference: ‐2.72; 95% CI:[‐4.84, ‐0.61]; *p* = 0.012). RT participants with poor sleep efficiency had better ADAS‐Cog 13 performance following the intervention (estimated mean difference: ‐2.63; 95% CI:[‐4.97, ‐0.28]; *p* = 0.029), compared with BAT participants with poor sleep efficiency. There were no effects of RT on ADAS‐Cog 13 for participants with good sleep duration or efficiency. Self‐reported sleep quality did not moderate intervention effects.

**Conclusion:**

RT appears to be particularly beneficial for cognitive function in adults with SIVCI and poor sleep.